# New sepsis-associated morbidity and mortality in pediatric oncology patients

**DOI:** 10.3389/fonc.2025.1638516

**Published:** 2025-08-27

**Authors:** Alicia M. Alcamo, Robert B. Lindell, Sydney A. Sheetz, Steven D. Ham, Andrew Strayer, Scott L. Weiss, Akira Nishisaki, Neethi P. Pinto, Alexis A. Topjian, Julie C. Fitzgerald

**Affiliations:** ^1^ Division of Critical Care Medicine, Department of Anesthesia and Critical Care, Children’s Hospital of Philadelphia and the Perelman School of Medicine, University of Pennsylvania, Philadelphia, PA, United States; ^2^ Pediatric Sepsis Program, Children’s Hospital of Philadelphia, Philadelphia, PA, United States; ^3^ Haverford College, Haverford, PA, United States; ^4^ Division of Critical Care, Department of Pediatrics, Nemours Children’s Health, Wilmington, DE, United States

**Keywords:** pediatrics, sepsis, oncology, critical care, morbidity

## Abstract

Sepsis is a leading cause of morbidity and mortality in children worldwide, yet the development of new morbidity after sepsis has not been clearly defined in high-risk subgroups such as children with cancer. Using the TOPICC (Trichotomous Outcome Prediction in Critical Care) multicenter cohort study dataset, we evaluated whether children with cancer have a higher risk of the composite outcome of death or new morbidity at hospital discharge compared to children without cancer. Among 854 children with sepsis, 88 patients (10.3%) had an underlying cancer diagnosis. Children with cancer were older (median 8.1 vs 3.7 years) and more frequently developed sepsis while in the hospital. The pattern of organ failure differed between groups, with less frequent invasive mechanical ventilation (26.1% vs 49.9%, *p*<0.001) but more frequent vasoactive infusions (47.7% vs 35.8%, *p*=0.03) in children with cancer compared to non-oncology patients. Children with cancer had an increased rate of death or new morbidity (22.7% vs 12.1%, *p=*0.006) compared to non-oncology patients. New morbidity (defined by ΔFSS score >2 points) occurred in 13.9% of cancer vs 6.9% of non-cancer survivors (*p*=0.03), and PICU mortality was similar between groups (10.2% vs 5.6%, *p*=0.09). Cancer diagnosis was independently associated with higher odds of death or new disability at discharge (adjusted odds ratio 3.71, *p*<0.001) in multivariable logistic regression, after adjusting for baseline FSS, baseline developmental delay, clinical concern for neurologic injury on PICU admission, and PICU supportive measures. These results suggest that children with cancer who develop sepsis are more likely to experience adverse outcomes at hospital discharge, even after accounting for baseline health and critical illness severity.

## Introduction

Sepsis remains a significant cause of childhood critical illness and death worldwide (1). In the United States alone, an estimated 72,000 children are hospitalized annually for sepsis (2), with approximately 9% experiencing mortality when admitted to the pediatric intensive care unit (PICU) ​ (3, 4). Children who survive sepsis frequently face new or worsened health issues after discharge, shifting the focus toward not only saving lives but also preserving long-term health-related quality of life (HRQL). Recent large-cohort analyses have shown that approximately 13% of children who survive critical sepsis develop a new chronic medical condition in the months after hospitalization (5)​. The most common new morbidities involve chronic respiratory failure, new dependence on supplemental feeding, and chronic kidney disease​ (5). These chronic health deficits suggest that many children who survive sepsis do not fully return to their pre-hospital baseline health status.

Beyond new chronic diagnoses, functional impairments and HRQL deterioration are recognized sequelae of sepsis. In the multicenter Life After Pediatric Sepsis Evaluation (LAPSE) study of 389 children admitted to the pediatric ICU for septic shock, 35% of survivors had a significant decline in HRQL from baseline that persisted for at least one year​ (6). Similarly, a report from the Outcomes Assessment Program at Seattle Children’s Hospital demonstrated that nearly one-quarter of children who survived sepsis failed to recover to their baseline HRQL, with almost one in four children showing persistent deficits​ (7). Notably, the study identified immune compromise or complex chronic disease as an independent risk factor for failure to regain baseline HRQL, indicating that children with pre-existing vulnerabilities, such as those undergoing cancer treatment, may be at especially high risk for long-term morbidity after sepsis (7). A separate analysis of hospital readmissions after sepsis found that immunocompromised children were frequently readmitted, often for infection-related complications​ (8). Taken together, these findings highlight that survival from sepsis is often just the beginning of a longer recovery trajectory, and that specific high-risk populations may experience disproportionate post-sepsis health burdens.

Children with cancer represent a unique subset of sepsis patients due to their immunosuppression, complex comorbidities, and ongoing therapies. Children with cancer are at high risk for life-threatening infections and sepsis, and infection remains a leading cause of death in this population, second only to progressive malignancy (9). Prior studies estimating the impact of cancer diagnosis on sepsis-associated mortality demonstrate mixed results, with some finding high mortality in children with cancer (10–13) but others finding similar mortality rates between children with and without cancer (14–16). Despite these studies, there is limited data specifically examining how sepsis affects functional outcomes in children with cancer. Given their underlying chronic illness, these patients may suffer higher rates of organ dysfunction during sepsis and may have reduced physiological reserve to recover from sepsis-associated critical illness. Additionally, clinicians may approach aggressive life-sustaining therapies differently in children with underlying malignancies, which could influence outcomes and functional recovery. Understanding how sepsis outcomes differ between children with and without cancer can inform prognostication, family counseling, and tailored post-PICU care for cancer survivors.

In this study, we leveraged data from a large multi-center cohort of children with sepsis to evaluate the association between a cancer diagnosis and functional outcomes at hospital discharge, specifically new morbidity or mortality. We hypothesized that children with cancer who develop sepsis have a higher risk of death or new morbidity compared to other children with sepsis after adjustment for severity of illness, and that this difference would be driven by differences in morbidity among survivors.

## Methods

We performed a secondary analysis of the Trichotomous Outcome Prediction in Critical Care (TOPICC) multicenter prospective observational study. TOPICC enrolled randomly selected children <18 years old admitted to the PICU from December 2011 to April 2013. A total of 10,078 children were enrolled at seven participating sites; only index PICU admissions were included (17). The Collaborative Pediatric Critical Care Research Network (CPCCRN) performed this study with support from the *Eunice Kennedy Shriver* National Institute of Child Health and Human Development (18). Institutional review board (IRB) approval was obtained at all participating sites. A de-identified dataset was obtained through a data use agreement with CPCCRN, and our secondary analysis was determined to be non-human subjects research by the Children’s Hospital of Philadelphia IRB.

### Study population

We identified our study population and exposures using primary or secondary PICU admission or discharge diagnoses in the TOPICC case report form. We restricted the initial TOPICC study population to those children with a primary or secondary PICU admission or discharge diagnosis of sepsis. Children were classified as having cancer, our primary exposure, if they had a primary or secondary PICU admission or discharge diagnosis of malignancy.

### Data variables

Demographic and clinical data available in the dataset were originally abstracted from the electronic health record at participating sites by trained study staff. Variables were selected *a priori* as potential factors associated with mortality and morbidity (17). Variables indicative of the severity of critical illness included the use of mechanical ventilation, vasoactive infusions, extracorporeal membrane oxygenation (ECMO) support, cardiopulmonary resuscitation (CPR), high-frequency oscillatory ventilation (HFOV), nitric oxide, renal replacement therapy (RRT), neuromuscular blockade, and steroid administration. Concern for acute neurologic injury by the clinical team at PICU admission was recorded, as well as the worst level of consciousness and pupillary response within 4 hours of PICU admission. The Functional Status Scale (FSS) was used to assess morbidity (19). Baseline FSS scores obtained in the primary study reflected pre-hospital functional status (17). Baseline Pediatric Cerebral Performance Category (PCPC) and Pediatric Overall Performance Category (POPC) scores were also obtained (20, 21).

Our primary outcome in this secondary analysis was a composite outcome comprised of death or new morbidity in survivors at hospital discharge. New morbidity was defined by an increase (worsening) of greater than 2 points in the FSS score from admission to discharge (17). Children with an increase of ≤2 points, no change, or an improvement in FSS score at discharge were classified as having no new disability. Death at hospital discharge and new morbidity at hospital discharge in survivors were evaluated individually as secondary outcomes.

The mode of death was evaluated for all nonsurvivors. Modes were characterized as failed resuscitation, withdrawal of technological support, limitation of care, and death by irreversible cessation of neurologic function. In addition to assessing overall change in FSS score, we also evaluated the change in FSS score for each FSS subdomain: mental status, sensory, motor, feeding, and respiratory.

### Statistical analysis

We first compared demographic and clinical characteristics for children with and without sepsis in the TOPICC cohort. Then, within the sepsis cohort, we compared demographic and clinical characteristics between children with and without cancer. Categorical data were expressed as frequencies and analyzed using Pearson’s chi-squared test. Continuous data were expressed as median with interquartile range (IQR) and analyzed using the Wilcoxon rank sum test. Logistic regression was used to create a bivariable base model with cancer diagnosis as the independent variable and the primary outcome, death or new morbidity, as the dependent variable. Multivariable logistic regression was used to test the association between oncological diagnosis and death or new morbidity at hospital discharge. Covariates that changed the odds ratio of the base model by more than 5% were included in the final model. We *a priori* included demographic and clinical variables (age, sex, history of any chronic conditions, history of developmental delay, admission source, and concern for acute neurologic injury at PICU admission) as potential covariates. We further examined the interaction between cancer diagnosis and the use of invasive mechanical ventilation or vasoactive infusions by including interaction terms (cancer × mechanical ventilation, cancer × vasoactive infusions) in the multivariable model. The multivariable model was created using stepwise backwards elimination with a *p*-value greater than 0.3 for the removal of variables. Because neurologic injury is a known risk factor for PICU morbidity and mortality, we performed a sensitivity analysis to evaluate outcomes between children with and without concern for acute neurologic injury at PICU admission. A *p*-value threshold of 0.05 was used to indicate statistical significance. Data were analyzed using Stata Version 18.5 (StataCorp, College Station, Texas).

## Results

Of the 10,078 children originally included in the TOPICC cohort, 854 children had sepsis as a primary or secondary diagnosis of cancer on PICU admission or discharge. Demographic and clinical characteristics of children with and without sepsis are shown in **Supplementary Table 1**. In comparison to children without sepsis, there was a higher proportion of children with cancer in the sepsis cohort (10.3% vs 6.0%, *p*<0.001). There was a higher proportion of children with baseline developmental delay in the sepsis cohort (35.4% vs 23.5%, *p*<0.001). Children with sepsis had a higher overall median baseline FSS score (7.0 vs 6.0, *p*<0.001) compared to those without sepsis.

Within the cohort of children with sepsis, we then compared demographic and clinical characteristics of children with and without cancer. Children with cancer were older (median age 8.1 years vs 3.7 years), were less frequently male (39.8% vs 55.9%), had a lower median baseline FSS score (6.0 vs 7.0), and less frequently had concern for acute neurologic injury at the time of PICU admission (6.8% vs 19.6%). Children with cancer were also more often admitted to the PICU from the general care floor, while children without cancer were more often admitted from the Emergency Department (**Table 1**). In comparing the severity of critical illness, children with cancer less frequently received mechanical ventilation (26.1% vs 49.9%, *p*<0.001) but more often were treated with vasoactive infusions (47.7% vs 35.8%, *p*=0.03) compared to those without cancer. There were no other statistical differences in other measured variables indicative of critical illness (**Table 2**).

**Table 1:** Demographic characteristics by presence or absence of cancer diagnosis.

Characteristic	Presence of cancer diagnosis (n=88)	Absence of cancer diagnosis (n=766)
Age, median [IQR]	8.1 (3.4-14.5)	3.7 (0.7-10.5)
Age group, n (%)		
0-1 years	8 (9.1)	309 (40.3)
2–5 years	29 (33.0)	150 (19.6)
6–12 years	22 (25.0)	159 (20.8)
13–18 years	29 (33.0)	148 (19.3)
Male sex, n (%)	35 (39.8)	428 (55.9)
Any Chronic Conditions, n (%)	83 (94.3)	550 (71.8)
Developmental Delay, n (%)	11 (12.5)	291 (38.0)
Congenital Heart Disease, n (%)	1 (1.1)	139 (18.1)
ICU admission source, n (%)		
Emergency Department	27 (30.7)	366 (47.8)
General Care Floor	44 (50.0)	114 (14.9)
Intermediate Care Unit	0 (0.0)	3 (0.4)
Operating Room	3 (3.4)	48 (6.3)
Outside Hospital	12 (13.6)	213 (27.8)
Other	2 (2.3)	22 (2.9)
Clinical concern for acute neurologic injury at ICU admission, n (%)	6 (6.8)	150 (19.6)
Worst LOC – within 4 hours of ICU admission, n (%)		
Coma/Unresponsive	2 (2.3)	57 (7.4)
No Coma	82 (93.2)	631 (82.4)
Unable to assess	4 (4.5)	77 (10.1)
Missing	0 (0.0)	1 (0.1)
Worst Pupillary response – within 4 hours of ICU admission, n (%)		
Both reactive	82 (93.2)	676 (88.3)
Both pupils < 3 mm, cannot be scored	1 (1.1)	54 (7.0)
One non-reactive (> 3 mm)	3 (3.4)	6 (0.8)
Both non-reactive (> 3 mm)	2 (2.3)	26 (3.4)
Missing	0 (0.0)	4 (0.5)
Baseline FSS, median [IQR]	6.0 (6.0-8.0)	7.0 (6.0-12.0)
Baseline PCPC, n (%)		
1 Normal	60 (68.2)	426 (55.6)
2 Mild Disability	21 (23.9)	122 (15.9)
3 Moderate Disability	7 (7.9)	80 (10.4)
4 Severe Disability	0 (0.0)	115 (15.0)
5 Coma/Vegetative	0 (0.0)	23 (3.0)
Baseline POPC, n (%)		
1 Good	17 (19.3)	266 (34.7)
2 Mild Disability	35 (39.8)	157 (20.5)
3 Moderate Disability	35 (39.8)	162 (21.1)
4 Severe Disability	1 (1.1)	158 (20.6)
5 Coma/Vegetative	0 (0.0)	23 (3.0)

IQR, interquartile range; ICU, intensive care unit; FSS, functional status scale; PCPC, pediatric cerebral performance category; POPC, pediatric overall performance category.

**Table 2:** Severity of critical illness by presence or absence of cancer diagnosis.

Exposure	Presence of cancer diagnosis (n=88)	Absence of cancer diagnosis (n=766)	P value
Mechanical Ventilation, n (%)	23 (26.1)	382 (49.9)	<0.001
HFOV, n (%)	4 (4.5)	25 (3.3)	0.53
Nitric Oxide, n (%)	4 (4.5)	43 (5.6)	0.68
Vasoactive Infusions, n (%)	42 (47.7)	274 (35.8)	0.03
ECMO, n (%)	0 (0.0)	18 (2.3)	0.15
CPR, n (%)	2 (2.3)	26 (3.4)	0.58
Renal Replacement Therapy, n (%)	4 (4.5)	36 (4.7)	0.95
Neuromuscular Blockade, n (%)	15 (17.0)	180 (23.5)	0.17
Steroids, n (%)	39 (44.3)	282 (36.8)	0.17

HFOV, high frequency oscillatory ventilation; ECMO, extracorporeal membrane oxygenation; CPR, cardiopulmonary resuscitation.

Children with cancer were more likely to experience our primary composite outcome of death or new morbidity at hospital discharge compared to those without cancer (22.7% vs 12.1%, *p*=0.01). Regarding our secondary outcomes, we did not identify a statistically significant difference in cohort mortality of children with and without cancer (10.2% vs 5.6%, *p*=0.09), though this comparison was limited by sample size (**Table 3**). For the children with cancer that died (n=9), withdrawal of technological support was the primary mode of death (78%, n=7) while the remainder died due to limitations of care (22%, n=2). Among children without cancer who died (n=43), withdrawal of technological support was the primary mode of death (63%, n=27), followed by failed resuscitation (21%, n=9), limitation of care (11%, n=5), and death by neurological criteria (5%, n=2). Within this limited sample, there was no statistical difference in the mode of death based on the presence of a cancer diagnosis. Among sepsis survivors, children with cancer were more likely to experience new morbidity compared to those without cancer (13.9% vs 6.9%, *p*=0.03; **Table 3**). In evaluating the change in FSS subdomain scores between the sepsis survivors with and without cancer, the feeding FSS subdomain was the only subdomain with a change that was different between children with and without cancer. Children with cancer were more likely to score >2 points higher (worse) in the feeding domain compared to those without cancer (25.3% vs 13.8%, *p*=0.01) (**Supplementary Table 2**).

**Table 3:** Primary and secondary outcomes by presence or absence of cancer diagnosis.

Outcome	Presence of cancer diagnosis (n=88)	Absence of cancer diagnosis (n=766)	P value
Death or new morbidity, n (%)	20 (22.7)	93 (12.1)	0.006
Hospital Mortality, n (%)	9 (10.2)	43 (5.6)	0.09
New Morbidity*, n (%)	11 (13.9)	50 (6.9)	0.04

*Denominator reflects survivors only (n=79 for children with cancer, n=723 for children without cancer).

In multivariable analysis, after controlling for severity of critical illness factors such as mechanical ventilation, vasoactive infusions, RRT, neuromuscular blockade and extracorporeal membrane oxygenation as well as baseline FSS score, presence of developmental delay, admission source, and concern for acute neurologic injury, children with cancer had increased odds of death or new morbidity at hospital discharge after sepsis compared to those without cancer (aOR 3.71, 95% CI 1.92-7.20, *p*<0.001) (**Table 4**). The interaction terms between cancer diagnosis and the use of mechanical ventilation and between cancer diagnosis and vasoactive infusions did not contribute to the final model following stepwise regression. Because mortality did not differ between groups, we separately tested the association between cancer diagnosis and new morbidity among survivors and found that children with cancer had increased odds of new morbidity at hospital discharge after sepsis compared to those without cancer (OR 2.18, 95% CI 1.08-4.38, p=0.03).

**Table 4:** Multivariable logistic regression for association between cancer diagnosis and death or new disability at hospital discharge.

Variable	aOR	95% CI	P value
Cancer Diagnosis			<0.001
No	Reference		
Yes	3.71	1.92-7.20	
Mechanical Ventilation			0.002
No	Reference		
Yes	2.88	1.49-5.56	
Vasoactive Infusions			0.01
No	Reference		
Yes	1.95	1.17-3.24	
Renal Replacement Therapy			0.004
No	Reference		
Yes	3.85	1.55-9.53	
Baseline FSS			0.002
No	Reference		
Yes	0.89	0.83-0.96	
Developmental Delay			0.12
No	Reference		
Yes	1.68	0.86-3.22	
Clinical concern for acute neurologic injury			<0.001
No	Reference		
Yes	3.34	1.93-5.78	
Neuromuscular Blockade			0.004
No	Reference		
Yes	2.36	1.32-4.22	
ECMO			0.07
No	Reference		
Yes	3.34	0.93-12.04	

aOR, adjusted odds ratio; CI, confidence interval; FSS, functional status scale; ECMO, extracorporeal membrane oxygenation.

Because concern for acute neurologic injury is a significant risk factor for PICU morbidity and mortality, we performed a sensitivity analysis evaluating outcomes for children with and without clinical concern for neurologic injury at PICU admission. Demographic characteristics of children in the study cohort with and without clinical concern for acute neurologic injury at PICU admission are shown in **Supplementary Tables 3, 4**. Among children with concern for acute neurologic injury at PICU admission, we found no difference in outcomes between children with and without a cancer diagnosis if there was a concern for neurologic injury. However, among children without concern for acute neurologic injury at PICU admission, children with cancer were more likely to experience death or new morbidity at hospital discharge compared to those without cancer (23.2% vs 9.4%, *p*<0.001; **Supplementary Table 5**).

## Discussion

In this secondary analysis of a large cohort of pediatric patients with sepsis, we found that children with cancer experienced significantly higher rates of new morbidity and mortality at hospital discharge compared to other pediatric sepsis patients. Importantly, having a cancer diagnosis was associated with adverse outcomes from sepsis even after controlling for baseline functional status and the severity of critical illness, suggesting that inherent factors related to malignancy may confer additional risk in the context of sepsis. To our knowledge, this is one of the first analyses to directly compare functional outcomes after sepsis between cancer patients and non-cancer patients. Our findings align with prior observations that immunocompromised status predicts poorer recovery after pediatric critical illness and underscore the unique vulnerability of immunocompromised children in the PICU​ (14, 22–24). We also observed that among children with sepsis who did not have concern for acute neurologic injury at PICU admission, children with cancer were more likely to experience new morbidity and mortality compared to those without cancer.

The association of clinical concern for acute neurologic injury at PICU admission with worse outcomes highlights the vulnerability of the brain in sepsis (25). Concern for neurologic injury was a robust predictor of adverse outcome in our study; a finding which aligns with prior work identifying neurologic organ dysfunction as a driver of long-term morbidity (26, 27). Children with sepsis-associated encephalopathy have been previously shown to have significant neurodevelopmental delays, reduced cognitive function after recovery, hospital readmission, and late mortality​ (28–31). However, although clinical concern for neurologic injury was a significant confounder for our multivariable model, when we performed a stratified analysis based on presence or absence of neurological injury on admission, the association of more frequent death or new disability in cancer patients compared to those without cancer persisted only in the group without neurological injury at admission. The strong association of clinical concern for acute neurological injury at presentation and poor outcomes likely outweighs the contributions of underlying comorbidities such as cancer to poor outcomes. Thus, our findings reinforce that preventing and mitigating acute neurologic injury in sepsis (for example, through aggressive management of shock and careful neuroprotective strategies) could be crucial for improving not just survival but also quality of life among all children with sepsis, irrespective of cancer status.

Several aspects of our results merit further discussion. First, we found that children with cancer had an increased odds of adverse outcomes following a PICU admission for sepsis, both looking at our primary composite outcome and then at new morbidity as a secondary outcome. While immunocompromised children with sepsis as a whole have an increased risk of mortality, several studies have shown no differences in hospital mortality for children with cancer (and without stem cell transplantation) compared to those without cancer after sepsis (15, 24, 32). This is in keeping with our results, in which we did not observe a statistically significant difference in mortality, but the findings of increased odds of the composite outcome of death or new morbidity may instead be driven by a higher frequency of new morbidity in patients with cancer. Indeed, we observed a 13.9% incidence of new functional disability among children with cancer who experienced sepsis, nearly double that of otherwise healthy children with sepsis. Prior studies have reported a wide range for new morbidity at discharge following pediatric sepsis, often around 10–25%, depending on the patient population and how disability is defined (5–7, 33–35). In our analysis, the rate of new morbidity in the non-cancer cohort falls at the lower end of that range, which may reflect the inclusion of a sizeable group of children with severe baseline disability in the cohort who were less likely to develop new morbidity according to our study definition (ΔFSS > 2). In contrast, children with cancer – who generally had good baseline performance status – were more susceptible to new deficits. Our findings are concordant with a recent report that 13% of pediatric sepsis survivors developed new chronic medical conditions after sepsis (5). Children with cancer are likely over-represented among those who develop such sequelae, given their predisposition to more severe and persistent organ toxicities. Our findings also align with data demonstrating higher rates of readmissions in children with cancer after sepsis (31).

Further, we found that children with cancer have a distinct organ failure profile during sepsis, with more shock and less respiratory failure. This is consistent with the typical sources of infection in immunocompromised hosts: central line-associated bloodstream infections and neutropenic sepsis may lead primarily to distributive shock, whereas previously healthy children often present with pneumonia leading to respiratory failure (36, 37). The lower rate of mechanical ventilation in the oncology group may reflect a true difference in sepsis epidemiology, but could also reflect differences in clinical decision-making related to code status. In our multivariable model, oncology patients who required mechanical ventilation had significantly higher mortality than those who did not, consistent with prior reports (38).

Our finding that nearly one in three children with cancer either died or had new morbidity at the time of discharge underscores the need for early and ongoing screening of functional status following hospitalization in sepsis survivors. Recent work from Goggin et al. (2024) has shown that in adult survivors of pediatric cancer, those who received treatment for sepsis during their cancer therapy were at increased risk for long-term neurocognitive impairments (39). Similar to children without cancer who survive sepsis, children with cancer are at increased risk for worse physical, emotional, cognitive, and social health outcomes. However, the significance of these long-term outcomes is most likely further exacerbated by the resumption of chemotherapy or other disease-directed treatments in children with cancer. Thus, children with cancer would benefit significantly from structured post-PICU follow-up to identify and manage new morbidities as they become evident (40, 41)​.

Strengths of this secondary analysis include the use of a large, multi-center cohort and the availability of granular functional outcomes data through the use of the FSS score. Despite these strengths, several limitations of our analysis are important to acknowledge. First, this is a retrospective secondary analysis of an existing dataset, and thus, we were limited by the variables collected by the TOPICC study and available in the de-identified dataset. We used new morbidity at hospital discharge to measure functional outcome, but this study cannot capture additional morbidity and mortality that develop after the initial hospitalization. Prior research has shown that pediatric sepsis survivors have substantial rates of hospital readmission and late mortality in the months following discharge. Thus, our findings might underestimate the full impact of sepsis on the trajectory of a child with cancer. Second, the sample of cancer patients with sepsis (n=88) is relatively small and heterogeneous in terms of cancer diagnoses, which limits our power to assess cohort mortality or to draw conclusions about specific subtypes of cancer or treatments and limits our ability to account for site-level variability in the sepsis care for this cohort. Third, although we adjusted for many confounders, unmeasured factors may influence outcomes. For instance, limitations in care were not explicitly captured; it is conceivable that these decisions would influence mortality independent of physiologic severity in a subset of pediatric oncology patients. Half of the children with cancer had been previously admitted to the hospital prior to the ICU admission for sepsis. Therefore, the change in FSS score may reflect parts of the hospitalization unrelated to sepsis. Conversely, highly aggressive interventions or investigational therapies might have been used in some patients and not others. Finally, advances in sepsis management and onco-critical care may have improved both short-term and long-term outcomes since the TOPICC study concluded in 2013. Nonetheless, the strong association we found between cancer status and worse outcomes suggests a meaningful difference that warrants ongoing attention.

In conclusion, children with cancer who develop sepsis have an increased risk for mortality or new functional morbidities at hospital discharge compared to other children with sepsis. For clinicians in the PICU, this knowledge can inform risk stratification: a child with cancer who develops sepsis should be recognized as having a high risk for adverse outcomes, prompting perhaps earlier involvement of multidisciplinary support during the PICU stay and consideration of post-discharge rehabilitation. For oncologists, awareness that a sepsis episode can significantly set back a child’s functional status may encourage closer monitoring for post-discharge morbidity. From a research standpoint, our findings support the need for further studies on recovery interventions tailored to immunocompromised pediatric patients.

## Data Availability

Publicly available datasets were analyzed in this study. This data can be found here: https://dash.nichd.nih.gov/study/226509.
