# Supplementary material for: New sepsis-associated morbidity and mortality in pediatric oncology patients
Source: Front Oncol. 2025 Aug 27;15:1638516. doi: 10.3389/fonc.2025.1638516 (PMC12420283; doi:10.3389/fonc.2025.1638516)
Supplement: Supplementary file 1 [file Table1.docx]

**SUPPLEMENTAL DIGITAL CONTENT**

**New sepsis-associated morbidity and mortality in pediatric oncology patients: a secondary analysis of the TOPICC dataset**

Alicia M. Alcamo^1,2 †^, Robert B. Lindell^1,2 †^, Sydney A. Sheetz^1^, Steven D. Ham^1,2^, Andrew Strayer^3^, Scott L. Weiss^4^, Akira Nishisaki^1^, Neethi P. Pinto^1,2^, Alexis A. Topjian^1^, Julie C. Fitzgerald^1,2*^

^1^ Division of Critical Care Medicine, Department of Anesthesia and Critical Care, Children’s Hospital of Philadelphia and the Perelman School of Medicine, University of Pennsylvania; Philadelphia, PA, USA.

^2^ Pediatric Sepsis Program, Children’s Hospital of Philadelphia; Philadelphia, PA, USA.

^3^ Haverford College; Haverford, PA, USA.

^4^ Division of Critical Care, Department of Pediatrics, Nemours Children's Health; Wilmington, DE, USA.

^†^These authors contributed equally to this work and share first authorship.

**Supplemental Table 1.** Demographic Characteristics for Admissions with or without Sepsis Diagnosis

| **Characteristic** | **Sepsis**  **(n=854)** | **No Sepsis**  **(n=9,224)** | **P-value** |
| --- | --- | --- | --- |
| **Age, median [IQR]** | 4.1 (0.9-11.7) | 3.7 (0.8-10.7) | 0.10 |
| **Age group, n (%)**   - 1. years   2-5 years  6-12 years  13-18 years | 317 (37.1)  179 (21.0)  181 (21.2)  177 (20.7) | 3,544 (38.4)  2,034 (22.1)  1,980 (21.5)  1,666 (18.1) | 0.28 |
| **Male sex, n (%)** | 463 (54.2) | 5,067 (54.9) | 0.69 |
| **Any Chronic Conditions, n (%)** | 633 (74.1) | 6,781 (73.5) | 0.70 |
| **Developmental Delay, n (%)** | 302 (35.4) | 2,169 (23.5) | <0.001 |
| **Congenital Heart Disease, n (%)** | 140 (16.4) | 2,422 (26.3) | <0.001 |
| **Cancer Diagnosis*, n (%)** | 88 (10.3) | 551 (6.0) | <0.001 |
| **Baseline FSS, median [IQR]** | 7.0 (6.0-11.0) | 6.0 (6.0-8.0) | <0.001 |
| **Baseline PCPC, n (%)**   1. Normal 2. Mild Disability 3. Moderate Disability 4. Severe Disability 5. Coma/Vegetative | 486 (56.9)  143 (16.7)  87 (10.2)  115 (13.5)  23 (2.7) | 6,473 (70.2)  1,437 (15.6)  672 (7.3)  564 (6.1)  78 (0.8) | <0.001 |
| **Baseline POPC, n (%)**   1. Good 2. Mild Disability 3. Moderate Disability 4. Severe Disability 5. Coma/Vegetative | 283 (33.1)  192 (22.5)  197 (23.1)  159 (18.6)  23 (2.7) | 3,633 (39.4)  3,019 (32.7)  1,680 (18.2)  811 (8.8)  81 (0.9) | <0.001 |

*=Includes all patients with cancer diagnosis listed at either ICU admission or discharge problem

IQR = interquartile range, FSS = functional status scale, PCPC = pediatric cerebral performance category, POPC = pediatric overall performance category

**Supplemental Table 2.** Worsening in Functional Status Scale (FSS) Scale Subdomain Scores by Presence of Cancer Diagnosis in PICU Survivors

| **Subdomain** | **Presence of Cancer Diagnosis**  **(n=79)** | **Absence of Cancer Diagnosis**  **(n=723)** |  | **P value** |
| --- | --- | --- | --- | --- |
| **Mental Status, n (%)** | 10 (12.6) | 56 (7.7) |  | 0.18 |
| **Sensory, n (%)** | 10 (12.6) | 50 (6.9) |  | 0.09 |
| **Motor, n (%)** | 16 (20.2) | 91 (12.6) |  | 0.09 |
| **Feeding, n (%)** | 20 (25.3) | 100 (13.8) |  | 0.01 |
| **Respiratory, n (%)** | 11 (13.9)) | 69 (9.5) |  | 0.29 |

Worsening in FSS domain score is defined as an increase in subdomain score by 2 or more points from baseline to hospital discharge.

**Supplemental Table 3.** Demographic Characteristics by Presence or Absence of Cancer Diagnosis for Children with Concern for Neurologic Injury on ICU Admission

| **Characteristic** |  | **Presence of Cancer Diagnosis**  **(n=6)** |  | **Absence of Cancer Diagnosis**  **(n=150)** |
| --- | --- | --- | --- | --- |
| **Age, median [IQR]** |  | 3.1 (2.3-4.6) |  | 3.7 (0.7-10.8) |
| **Age group, n (%)**  0-1 years  2-5 years  6-12 years  13-18 years |  | 1 (16.7)  4 (66.6)  1 (16.7)  0 (0.0) |  | 62 (41.3)  27 (18.0)  32 (21.3)  29 (19.3) |
| **Male sex, n (%)** |  | 1 (16.7) |  | 92 (61.3) |
| **Any Chronic Conditions, n (%)** |  | 6 (100.0) |  | 108 (72.0) |
| **Developmental Delay, n (%)** |  | 3 (50.0) |  | 89 (59.3) |
| **Congenital Heart Disease, n (%)** |  | 0 (0.0) |  | 28 (18.7) |
| **Baseline FSS, median [IQR]** |  | 9.0 (6.0-11.0) |  | 12.0 (6.0-18.0) |
| **Baseline PCPC, n (%)**   1. Normal 2. Mild Disability 3. Moderate Disability 4. Severe Disability 5. Coma/Vegetative |  | 1 (16.7)  4 (66.6)  1 (16.7)  0 (0.0)  0 (0.0) |  | 55 (36.7)  12 (8.0)  22 (14.6)  49 (32.7)  12 (8.0) |
| **Baseline POPC, n (%)**   1. Good 2. Mild Disability 3. Moderate Disability 4. Severe Disability 5. Coma/Vegetative |  | 1 (16.7)  2 (33.3)  2 (33.3)  1 (16.7)  0 (0.0) |  | 44 (29.3)  13 (8.7)  24 (16.0)  57 (38.0)  12 (8.0) |

IQR = interquartile range, FSS = functional status scale, PCPC = pediatric cerebral performance category, POPC = pediatric overall performance category

**Supplemental Table 4.** Demographic Characteristics by Presence or Absence of Cancer Diagnosis for Children without Concern for Neurologic Injury on ICU Admission

| **Characteristic** | **Presence of Cancer Diagnosis**  **(n=82)** | **Absence of Cancer Diagnosis**  **(n=616)** |
| --- | --- | --- |
| **Age, median [IQR]** | 9.2 (3.6-14.6) | 3.8 (0.7-10.5) |
| **Age group, n (%)**  0-1 years  2-5 years  6-12 years  13-18 years | 7 (8.5)  25 (30.5)  21 (25.6)  29 (35.4) | 309 (40.3)  150 (19.6)  159 (20.8)  148 (19.3) |
| **Male sex, n (%)** | 34 (41.5) | 428 (55.9) |
| **Any Chronic Conditions, n (%)** | 77 (93.9) | 550 (71.8) |
| **Developmental Delay, n (%)** | 8 (9.8) | 291 (38.0) |
| **Congenital Heart Disease, n (%)** | 1 (1.2) | 139 (18.1) |
| **Baseline FSS, median [IQR]** | 6.0 (6.0-7.0) | 7.0 (6.0-12.0) |
| **Baseline PCPC, n (%)**   1. Normal 2. Mild Disability 3. Moderate Disability 4. Severe Disability 5. Coma/Vegetative | 59 (72.0)  17 (20.7)  6 (7.3)  0 (0.0)  0 (0.0) | 371 (60.2)  110 (17.9)  58 (9.4)  66 (10.7)  11 (1.8) |
| **Baseline POPC, n (%)**   1. Good 2. Mild Disability 3. Moderate Disability 4. Severe Disability 5. Coma/Vegetative | 16 (19.5)  33 (40.2)  33 (40.2)  0 (0.0)  0 (0.0) | 222 (36.0)  144 (23.4)  138 (22.4)  101 (16.4)  11 (1.8) |

IQR = interquartile range, FSS = functional status scale, PCPC = pediatric cerebral performance category, POPC = pediatric overall performance category

**Supplemental Table 5.** Outcomes by Presence or Absence of Cancer Diagnosis and Concern for Neurologic Injury on Admission

| **Outcome** |  | **Presence of Cancer Diagnosis** |  | **Absence of Cancer Diagnosis** |  | **P value** |
| --- | --- | --- | --- | --- | --- | --- |
| **Concern for Neurologic Injury** |  | (n=6) |  | (n=150) |  |  |
| **Death or new morbidity, n (%)** |  | 1 (16.7) |  | 35 (23.3) |  | 0.70 |
| **Hospital Mortality, n (%)** |  | 1 (16.7) |  | 20 (13.3) |  | 0.81 |
| **New Morbidity*, n (%)** |  | 0 (0.0) |  | 15 (11.5) |  | 0.42 |
| **No Concern for Neurologic Injury** |  | (n=82) |  | (n=616) |  |  |
| **Death or new morbidity, n (%)** |  | 19 (23.2) |  | 58 (9.4) |  | <0.001 |
| **Hospital Mortality, n (%)** |  | 11 (13.4) |  | 35 (5.7) |  | 0.008 |
| **New Morbidity^, n (%)** |  | 8 (11.3) |  | 23 (4.0) |  | 0.006 |

*=Denominator reflects survivors only (n=5 for children with cancer, n=130 for children without cancer); ^=Denominator reflects survivors only (n=71 for children with cancer, n=581 for children without cancer)
